# A Systematic Review on the Role of Wildlife as Carriers and Spreaders of *Campylobacter* spp.

**DOI:** 10.3390/ani13081334

**Published:** 2023-04-13

**Authors:** Andrea Margarita Olvera-Ramírez, Neil Ross McEwan, Karen Stanley, Remedios Nava-Diaz, Gabriela Aguilar-Tipacamú

**Affiliations:** 1 Cuerpo Académico Salud Animal y Microbiología Ambiental, Facultad de Ciencias Naturales, Universidad Autónoma de Querétaro, Avenida de las Ciencias S/N, Juriquilla, Delegación Santa Rosa Jáuregui, Querétaro C.P. 76230, Mexico; 2 School of Pharmacy and Life Sciences, Robert Gordon University, Aberdeen AB10 7GJ, UK; 3 Department of Biosciences and Chemistry, Sheffield Hallam University City Campus, Howard Street, Sheffield S1 1WB, UK; 4 Posdoctoral CONACyT Program, Facultad de Ciencias Naturales, Universidad Autónoma de Querétaro, Avenida de las Ciencias S/N, Juriquilla, Delegación Santa Rosa Jáuregui, Querétaro C.P. 76230, Mexico

**Keywords:** *Campylobacter*, wildlife, sources

## Abstract

**Simple Summary:**

Wildlife are important reservoirs of bacterial pathogens associated with human diseases. Campylobacteriosis is a relevant gastrointestinal disease in humans and is caused principally by *Campylobacter jejuni* and *Campylobacter coli*. This review compiles the current knowledge of the potential for wildlife to carry and spread *Campylobacter* spp.

**Abstract:**

*Campylobacter* spp. are important zoonotic pathogens and can cause one of the main bacterial diarrheal diseases worldwide. Research in the context of infection arising from transmission from other humans and other vertebrates has been extensive. A large fraction of these investigations has focused on domestic animals; however, there are also a number of publications which either totally, or at least in part, consider the role of wild or feral animals as carriers or spreaders of *Campylobacter* spp. Here, we carry out a systematic review to explore the role played by wild vertebrates as sources of *Campylobacter* spp. with a compilation of prevalence data for more than 150 species including reptiles, mammals and birds. We found that numerous vertebrate species can act as carriers of *Campylobacter* species, but we also found that some host specificity may exist, reducing the risk of spread from wildlife to domestic animals or humans.

## 1. Introduction

Bacterial species of the genus *Campylobacter* include zoonotic pathogens, some of which can be emergent and highly pathogenic [1]. Human campylobacteriosis, the infection caused by members of the genus *Campylobacter*, manifests as gastroenteritis and is one of the four leading causes of diarrheal diseases worldwide [2]. Also, severe neuropathological disorders Guillain–Barré syndrome (GBS) and Miller Fisher syndrome (MFS), and reactive arthritis have been associated with *Campylobacter* [3].

Despite human campylobacteriosis mainly being caused by *Campylobacter jejuni* and *Campylobacter coli* [4], a broad range of other *Campylobacter* spp. have also been isolated from human clinical samples including: *Campylobacter lari*, *Campylobacter fetus*, *Campylobacter concisus*, *Campylobacter rectus*, *Campylobacter mucosalis*, and *Campylobacter upsaliensis* [5,6]. 

Domestic and companion animals, livestock, and several species of laboratory animals can also become infected with *Campylobacter* spp. [7,8,9,10,11,12,13,14]. In addition, *Campylobacter* spp. have been isolated from the intestinal tracts of a wide variety of healthy and diseased mammals and birds, including poultry, ruminants, and swine [15,16,17,18,19,20]. Therefore, animals are considered as reservoirs of these bacteria because zoonotic transmission of *Campylobacter* is thought to occur predominantly from contact with infected livestock and poultry [21,22,23]. However, wildlife can be reservoirs, sources or amplifying hosts [24], in that they provide a high pathogen-shedding capacity and may play an important role in the transmission of zoonotic pathogens. While there are several reports of the presence of *Campylobacter* species in wild mammals, many of these reports act as individual papers, almost in the form of case studies, or have concentrated on their impact on domesticated animals with a view to a potential impact on human populations. Here, we draw together data from these papers to systematically evaluate the diversity of vertebrate species, with an emphasis on those found in the wild, which have been shown to have been infected by *Campylobacter* with the objective of giving a more complete understanding of the range of vertebrate species known to have been identified as being infected by *Campylobacter*.

## 2. *Campylobacter*-Associated Pathogenesis in Humans

*Campylobacter* spp. are part of the *Campylobacteriaceae* family. These bacteria are Gram-negative rods, small (0.2–0.9 μm wide and 0.2–5.0 μm long), spirally curved, and do not form spores. They move in a way that resembles a corkscrew [25,26,27] and are chemoorganotrophs and obtain their energy sources from amino acids or tricarboxylic acid cycle intermediates [28]. The genus *Campylobacter* consists of 32 officially described species and 9 subspecies [29]. 

*Campylobacter* is the most reported cause of bacterial infectious gastrointestinal disease. However, systematic disease surveillance programs, which include campylobacteriosis, are largely limited to industrialized countries, such as the United States and member states of the European Union, because in non-industrialized countries they are either scarce or have a lower incidence [26,30]. *Campylobacter* infections in humans principally cause diarrhea; however, the severe neuropathological disorders Guillain–Barré syndrome (GBS) and Miller Fisher syndrome (MFS), and reactive arthritis have been associated with *Campylobacter* infections [3,31].

*C. jejuni* was first identified as a human diarrheal pathogen in 1973 [32]. The major relevance of campylobacters as a main cause of human disease was just uncovered in the early 1980s. The pathogenesis of *C. jejuni* infection involves both host- and pathogen-specific factors [32]. This bacterium can affect people of all ages but with distinctive bimodal distribution, affecting children aged <4 years and people aged 15–44 years, also individuals with AIDS [26].

Campylobacteriosis is the most common disease caused by *Campylobacter* spp. These bacteria have a worldwide distribution and a wide host variability. Food-producing animals such as cattle, sheep, swine, and poultry commonly harbor *Campylobacter* spp. in their gastrointestinal tracts [17,33,34] and represent an important route through which organisms could enter the food *chain*.

Aquatic birds are reservoirs of many *Campylobacter* spp. such as *C. jejuni* and *C. coli* [35]. However, it has also been suggested that wild birds are carriers of *Campylobacter* spp. and a source of infection for other species of animals and humans [36]. Kwan et al. [37] reported molecular evidence, MLST among *C. jejuni* isolates (*n* = 130; 59 from humans, 40 from raw peas, and 31 from wild birds) of an outbreak, and demonstrated the association of many more human *C. jejuni* infections associated with the outbreak than with raw peas or wild bird feces.

On the other hand, the pattern and distribution of *C. jejuni* infection differs from wild free-ranging animals to domestic ones [38]. A study identified 443 isolates of *C. jejuni* and *C. coli* in stools of 2031 domestic animals such as cattle, sheep, and pigs, as well as birds and pets [39]. The prevalence was generally between 22 and 28%, and there was a higher prevalence in poultry (41%) than in cats and dogs (<5%). Moreover, using MLST, it was demonstrated that there is a host specificity for infection [39]. 

Various routes of transmission of *Campylobacter* spp. have been described. One such example is that it has been suggested that the supply of water is a determining factor in transmission, as Shrestha et al. [40] showed that *Campylobacter* spp. have been isolated from recreational rivers. The strains isolated were generally associated with wild birds but also occasionally associated with human diseases. 

Other authors have studied the presence and diversity of virulence-associated genes among *Campylobacter* strains isolated from wild birds as complementary evidence to their role in the epidemiology of human campylobacteriosis. DNA extraction and amplification have targeted several virulence-associated genes including those related to adhesion and colonization (*cadF*), invasion (*ciaB*, *virB11*, *htrA*, and *hcp*), cytolethal distending toxin (*cdtA*, *cdtB*, *cdtC*), and flagellin (*flaA* and *flaB*) genes [41,42,43]. Additionally, the ability to invade human colonic epithelial cells has been tested through the gentamicin protection assay [42].

## 3. Literature on Wildlife Carriers of *Campylobacter* spp.

Literature was searched on the ISI—Web of Science and PubMed databases on September 26, 2022, using the terms: (Campylobacter*) AND (wildlife OR amphibian* OR* fish OR reptile* OR bird* OR mammal*) AND (reservoir OR prevalence OR maintenance). This systematic review followed the Preferred Reporting Items for Systematic Reviews and Meta-Analyses (PRISMA) guidelines. Journal articles or short communications were selected with no restriction on the publication date, while books and book chapters were excluded since they were unlikely to be primary research publications.

All duplicate reports were removed. The title and abstract from selected reports were scanned and these were included if: (i) they reported information on free-living wildlife or individuals captured or recently admitted to rehabilitation centers, (ii) documented findings on any *Campylobacter* species, (iii) data were obtained through an observational study. Papers were excluded if: (i) they only reported information on domestic or captive animals unless they were to be included for the purposes of illustrating that infection had been reported in that species, (ii) data were obtained through experimentation, or (iii) they were summaries, reviews, or meta-analyses, or (iv) full text was not available. After this first round of selection, we carried out a second round based on a full reading of the articles. This resulted in the dismissal of additional reports whose selection was not straightforward based on title and abstract screening. Using this web-based review search, 245 papers in Web of Science and 199 in PubMed were identified. Four publications were excluded, because they were not scientific articles. Once the results were pooled and duplicates were eliminated, 306 unique publications ranging from the years 1981 to 2022 remained. Initial scanning of the title and abstract showed that 118 articles did not meet the inclusion criteria. Five articles were excluded because neither the abstract nor the full text were available. Also, five articles were dismissed, because they analyzed data reported in previous publications that were selected in this review. Full reading of the text resulted in the dismissal of twelve articles based on them not providing any new information in the context of the purpose of this review. A final selection of 166 articles remained for comprehensive revision.

## 4. Wildlife Sources of *Campylobacter* Species

The degree of similarity between *Campylobacter* isolates found in infected humans and wild birds is a widely studied topic. Studies aimed to discriminate among isolates from wild birds and humans have included samples from water, soil, and, to a lesser extent, poultry samples. However, in terms of the current paper, we list below some of the methods which have been used as an approach to confirming the presence of *Campylobacter* spp. in samples.

*Campylobacter* spp. can be isolated from several samples including stools, rectal, and blood samples [26], using either selective or nonselective medium followed by an incubation period in a microaerobic atmosphere. Antibiotics may be used to suppress other microbiota growth [44,45]. Furthermore, microscopic examination of colonies requires Gram staining, a motility test, and an oxidase test [26]. Serological methods, such as passive hemagglutination and latex agglutination, are used to detect *Campylobacter* spp. [46,47]. However, molecular typing methods have largely replaced serological ones due to their increased availability and discriminatory power. Such methods have been employed in source attribution, isolates discrimination [6,48,49], and in the control of foodborne pathogens interventions [50].

Most of the molecular analytical tools have been developed for work with either medical reasons or for work with domesticated animals. For example, *C. jejuni* and *C. coli* isolates from poultry, cattle, and humans have been studied using different approaches, including both pulsed-field gel electrophoresis (PFGE) and PCR of candidate marker genes [51,52]. In addition, multiplex PCR has been used for identification and differentiation of the thermophilic species *C. jejuni* and *C. coli*, principally in poultry samples [53] although, Backhans et al. [54] used the same primers for detection in wild rodents, meaning that although this approach was developed for domesticated animals, it has been shown to be equally useful in wild animals. Furthermore, multilocus sequence typing (MLST), a technique that determines the sequence diversity of multiple loci which characterize isolates of microbial species using the DNA sequences of internal fragments of multiple housekeeping genes, has been employed, e.g., *flaA* SVR typing [55] and the ST-45 and ST-677 complexes [56]. With regards to *Campylobacter*, this technique has been used initially to determine sequences of *C. coli* in pig liver, as well as human, poultry, and bovine isolates [55,56]. In the wildlife context, this approach has been used to detect *C. jejuni* isolates in wild birds and rabbits [36,37]. Also, using MLST and phylogenetic analysis has provided evidence that some strains isolated from wild birds can be shared with humans, domesticated birds in the form of poultry, and livestock, while other strains detected form separate groups, because they differ to a larger extent from strains isolated from humans and domestic animals [41,49].

## 5. Wildlife Carriers of *Campylobacter* Species

**Figure 1:**
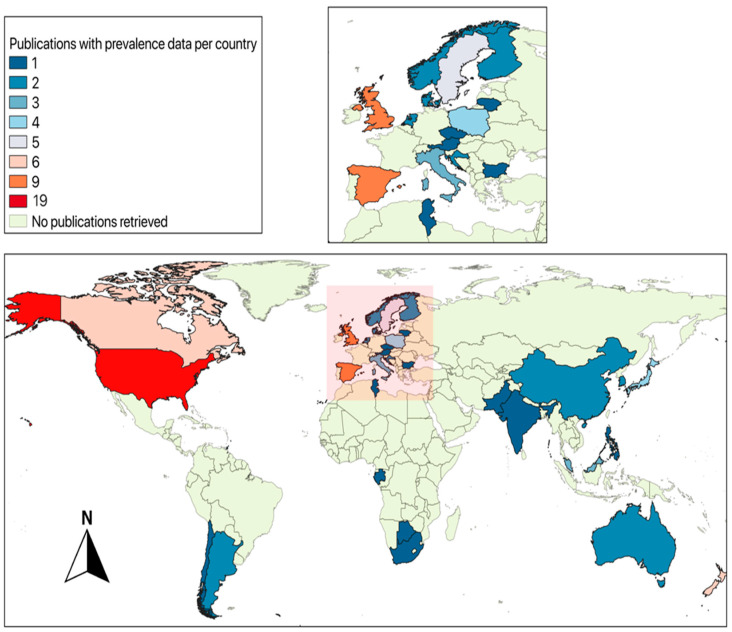
Global map (made it with QGIS 3.4 software [149]) representing the number of publications with *Campylobacter* prevalence in wildlife per country. The number of publications per country are color-coded as per the index and the top left of the figure. The area shaded in the box at the bottom of the figure is shown in more detail in the top right corner of the figure due to the close geographical proximity of many of the European nations in which papers on the topic have been published.

Data from the articles reviewed showed that at least twelve *Campylobacter* species have been detected in wild animals in 36 countries and the Antarctica Peninsula [15,16,38,40,41,42,43,46,48,49,50,57,58,59,60,61,62,63,64,65,66,67,68,69,70,71,72,73,74,75,76,77,78,79,80,81,82,83,84,85,86,87,88,89,90,91,92,93,94,95,96,97,98,99,100,101,102,103,104,105,106,107,108,109,110,111,112,113,114,115,116,117,118,119,120,121,122,123,124,125,126,127,128,129,130,131,132,133,134,135,136,137,138,139,140,141,142,143,144,145,146,147,148] (Figure 1). Details of the animal species involved are found in Table S1 in the supplementary data. The most commonly detected species was *C. jejuni*, followed by *C. coli* and *C. lari*. However, other species, *C. fetus*, *Campylobacter helveticus*, *C. upsaliensis*, *Campylobacter hyointestinalis*, *Campylobacter sputorum*, *Campylobacter canadensis*, *Campylobacter hepaticus*, *Campylobacter subantarcticus*, and *Campylobacter volucris* have also been sporadically detected in wildlife. Prevalence estimates for reptiles, mammals, and birds species are presented in Table S1, and this includes information for *Campylobacter*, *C. jejuni*, *C. coli*, and *C. lari* when available.

### 5.1. Fish, Amphibians, and Reptiles 

In general, most people consider *Campylobacter* infections as being a problem associated with homeotherms due to their body temperature being maintained at a level which is conducive to the growth of *Campylobacter* spp. However, there are several examples of *Campylobacter* infections being documented in other vertebrate species. *Campylobacter* have been described more widely in a range of different Squamates, as reviewed previously [150].

The species identified in squamates have included two subspecies of *C. fetus* (*C. fetus* subspecies *fetus* and *C. fetus* subspecies *testudinum*), *C. jejuni*, and *Campylobacter iguaniorum*. These have all been seen in reptiles; primarily lizards such as geckos and iguanas, and also in some species of snakes [151]. Examples of lizards with infections have been seen in both Europe [152] and Australia [153]. The work of Gilbert et al. [152] included detection of *C. fetus*, *C. hyointestinalis*, and *Campylobacter* spp. by both cultivation and PCR approaches. In each case, the PCR approach had a higher detection rate than that using cultivation as follows: lizards (62% versus 11%), snakes (32% versus 3%), and turtles (93% versus 39%). It is also worth noting that turtles also had the highest infection rates for two other genera of bacteria: *Arcobacter* and *Helicobacter*.

A study in Taiwan detected *C. fetus* in both wild and domesticated reptiles [150], with *C. fetus* a species which was also shown to be able to cross into the human population [154]. Although *C. fetus* has been described in both reptiles and mammals, there appears to be host dichotomy between species, with genetic divergence between the lineages in mammals and reptiles [155]. Although *Campylobacter* infections in reptiles have primarily been described in squamates, there are also examples of infection in other reptiles, such as chelonians. One such example is from red-footed tortoises (*Chelonoidis carbonaria*) in captivity [155] with other reports in turtles [151]. However, no evidence of *Campylobacter* infection was found in European pond turtle (*Emys orbicularis*) and read eared slider (*Trachemys scripta elegans*) [156].

There are also examples of *Campylobacter* infections in fish. For example, *C. cryaerophila* has been isolated from rainbow trout (*Oncorhynchus mykiss*) [157] and also a study using other freshwater fish (*Capoeta capoeta capoeta, Capoeta trutta, Alburnoides bipunctatus*, and *Leuciscus cephalus*) [158]. However, as reported by Loewenhwerz-Lüning et al. [159], the incidence of infection was much lower in fish than that seen in homeotherms, with many investigations failing to either cultivate *Campylobacter* from fish samples or to detect members of this genus by PCR.

In the remaining class of non-homeothermic vertebrates (amphibians), reports detecting *Campylobacter* are scarce in the literature. The reports do exist, such as when *Campylobacter*-like bacteria were described in frogs in the early 1980s [160], and there has been a *C. fetus* infection arising from meals which included consumption of frog meat [158]. However, in several other pieces of work, it was not possible to detect *Campylobacter* by either culturing methods or using PCR (e.g., Martel et al. [161]).

### 5.2. Birds 

Despite much of the research carried out on *Campylobacter* species involving mammals, the importance of infection in birds cannot be underestimated. Much of this is down to the fact that they have a body temperature which is ideal for *Campylobacter* to proliferate [162]. This is true for both domesticated poultry [163] and also wild birds [162,163]. Particularly in the case of wild birds, this is problematic as their ability to fly means that they have the potential to spread *Campylobacter*, as well as other zoonotic organisms, by crossing over geographical barriers [164]. 

In a study of microbial infection in several vertebrate species [165], it was shown that 17% of cloacal samples collected from wild birds were positive for *Campylobacter*, but <1% of racing pigeons were infected. This was the converse of observations for *Salmonella*, where <1% of the wild birds were infected, but 5% of the racing pigeons were infected. This may suggest that different enteropathogens are more prevalent in the wild population relative to those in captivity.

However, there can be considerable variation in terms of the levels of infection observed, even within a single country. For example, in Scotland pheasants are often bred in captivity prior to being released into the wild for sporting purposes. There they will come into contact with a population of wild pheasants (*Phasianus colchicus*) which is present as well. Thus, the Scottish pheasant population can be thought of as being both wild and also semiferal. Based on previous work [143,166,167,168,169], Seguino et al. [170] predicted that around a quarter of wild pheasants would be infected with at least one species of *Campylobacter*. However, after sampling from 5 different parts of Scotland, it was found that over 36% of the birds were positive, ranging from 50% in the Borders to only 6.8% in the Southwest of the country. When data were examined for each individual estate sampled, there was even greater variation, with one estate in the Borders having 73.3% infection, and one of the estates in the Glasgow area having no infections detected, reiterating the point that there can be considerable geographical variation, even for samples collected relatively close together. 

Detection of prevalence estimates are commonly reported for single species. However, Konicek et al. [143] presented the percentage of birds positive for *Campylobacter* for each order of birds, even though sample size was extremely uneven across orders. The largest proportions of positive samples were detected for Anseriformes, Passeriformes, Charadriiformes, Gruiformes, and Columbiformes.

Several studies have addressed *Campylobacter* prevalence in bird species whose ecological habitats increase their infection risk and transmission potential [35,48,140]. Species such as herring gulls (*Larus argentatus*), rock pigeons (*Columba livia*), American crows (*Corvus* spp.), and European starlings (*Sturnus vulgaris*) have repeatedly been monitored due to their feeding habits and their close contact with human populations. For example, herring gulls, which are opportunistic scavengers, can use human waste as food, while European starlings and American crows can forage and roost in agricultural and urban areas.

Irrefutable data are not available to support the hypothesized role of synanthropic birds as relevant *Campylobacter* sources for transmission to humans. However, studies focusing on pigeons and doves showed that a large number of the birds sampled tested negative without evidence of infection, ranging between 75% (18/24) and 91% (98/107) being negative [42,140].

Current information shows that the prevalence of *Campylobacter* in crows can vary widely, and, more importantly, it suggests that crows are frequently infected with *Campylobacter*. Prevalence data are available for *Campylobacter macrorhynchos* (19.4%, 27/139), *Campylobacter brachyrhynchos* (66.9%, 85/127), and *Campylobacter monedula* (100%, 4/4) [62,63,64]. Results published therein indicate a sharp predominance of *C. jejuni* among the isolates (above 90% in all cases).

As mentioned earlier, geographical barriers can pose less of a challenge to birds, relative to other animals. This is particularly true for migratory species, with many species migrating thousands of miles twice a year. Specifically, for those which migrate longer distances, there are often key stopping off points during their migration for resting, feeding, etc. In many species, this happens in countries which have a border with the Mediterranean Sea. These provide biannual areas where migrant birds can either infect, or become infected by, the resident population. One such country is Turkey, which is a key stopping point for many species of birds and provided a site for a recent survey of infection of birds by *Campylobacter* [35]. In this work, three of the five species (turtle doves (*Streptopelia turtur*), red-crested pochards (*Netta rufina*), and quails (*Coturnix coturnix*)) which were sampled failed to have any *Campylobacter* detected, while the other two species showed widely different infection levels (93% in coots (*Fulica atra*) but only 5.2% in song thrushes (*Turdus philomelos*). This suggests that landing in this area has the potential to expose migratory birds to other infected species of birds, but that there is great interspecies variation in the infection rates. Therefore, bird migratory behavior is potentially considered a relevant factor in the spread of *Campylobacter* and other pathogenic microorganisms over large distances due to carriage by a suitable host. This issue has been addressed with some groups such as shorebirds, gulls, ducks, rails, raptors, and songbirds [64,141,171]. For example, Ryu et al. [141] identified high numbers of *Campylobacter*, including *C. lari*. This work involved stool samples collected from examples of the shorebird species red knot (*Calidris canutus*), semipalmated sandpiper (*Calidris pusilla*), and ruddy turnstone (*Arenaria interpres*) which showed the presence of *Campylobacter.* However, neither *C. jejuni* nor *C. coli* were detected, but, rather, *Campylobacter lari* was present [167]. In contrast, *C. jejuni* was the predominant species in samples obtained from migratory passerines of the Paleartic (36/39 of samples positive for *Campylobacter*) [64]. Overall prevalence of *Campylobacter* was low to moderate in this group since prevalence for long-distance migrants and short-distance migrants was 17.2% (17/99) and 31.5% (22/70), respectively. Therefore, birds must be treated as important potential spreaders of *Campylobacter* infection, particularly due to the ability of birds to cross geographical barriers, although sampling sites and species sampled play an important role in analysis. 

Overall, *Campylobacter* isolates from wild birds harbor major virulence-associated genes [172]. However, not all bird species and isolated strains seem to play a significant role in human infection because of the low prevalence of virulence-associated genes. Weis et al. [41] reported the presence of the CDT (cytolethal distending toxin) gene cluster in 20% of the *C. jejuni* isolates obtained from crows, while Iglesias-Torrens et al. [49] found that 46% of the wild bird strains, including storks, ravens, pigeons, and gulls, tested negative for at least one of the *cdt* genes. In the case of *C. jejuni* isolates obtained from crows, Weis et al. [41] reported the presence of the CDT gene cluster in 20% of the samples. The same gene cluster was present in 92% and 100% of the crow isolates obtained from Washington, USA, and Kolkata, India, respectively [158]. However, these isolates had a truncated gene cluster, meaning that these bacteria could not produce a functional toxin protein. Shyaka et al. [42] scanned isolates from different species, including crows and pigeons, for the presence of 7 virulence-associated genes. Only 21% (7/33) of the samples harbored all the genes studied, while 75% (25/33) of crow and Eurasian tree sparrow (*Passer montanus*) isolates were positive for all the genes tested other than *cdtA*.

In addition to direct transmission between, and within, species of birds, there are reports of house flies (*Musca domestica*) being possible vectors for the spread of *Campylobacter*, with flies which had become inoculated having live *Campylobacter* for up to 24 h after inoculation. Interestingly, the bacteria which were still viable after 24 h were the ones in flies which were kept at 15 °C, whereas those which were in flies at typical temperatures seen in homotherms such as birds could rarely, if ever, be detected after 24 h [173].

Thus, birds not only provide a potential for transmission of *Campylobacter* directly in the wildlife and domestic animals but may also allow for indirect transmission via flies as an intermediate. In addition, work with *Campylobacter* has been used as a model system in the endangered New Zealand bird species; the takahe (*Porphyrio hochstetteri*) [174]. In this species, it has been shown that 99% of the birds harbored one or more species. In addition to *C. jenuni* (present in 38% of the birds examined) and *C. coli* (24%), there was also around 90% prevalence of *Campylobacter* sp. *nova 1*, which has only been detected in New Zealand. Thus, this has been proposed as a model for the interaction between hosts and pathogens in an isolated population.

#### Potential Role of Birds in Spreading Antibiotic Resistance via *Campylobacter* spp.

The ability for birds to spread *Campylobacter* becomes even more of a concern when it is noted that this can include strains which have antibiotic resistance genes [175]. In a recent survey [162] of cloacal swabs, it was found that almost a third of wild waterfowl were carriers of *Campylobacter* species, with four of the five species harboring *C. jejuni* and mallards also carrying *C. coli*. All 30 samples tested positive for several different virulence genes, with 11 of them also having one or more genes for antibiotic resistance present. Given that these waterfowl species are often found on farmland, or water which runs through farmland, they pose a risk to farm animals, both in terms of the spread of *Campylobacter* per se, but also the spread of antibiotic resistance genes.

By way of further illustrating this, it is worth noting that Sippy et al. [176] reported that birds play an important role in the epidemiology of pathogenic *Campylobacter* and can act as a reservoir for antibiotic-resistant *Campylobacter* which can infect livestock. Overall, *Campylobacter* spp. prevalence was 4.79% (9/188), and of the 9 isolates, 22.2% were *C. coli* and 77.7% were *C. jejuni*, and most *Campylobacter* isolations (5/9; 55.6%) were from white-throated sparrows (*Zonotrichia albicollis*). 

As already noted, *Campylobacter* were found in all crows studied in one particular study [43] but were absent from gulls in another study [49]. These are birds which have a reputation as scavengers and so will be likely to be exposed to a range of different food sources. However, they are not counted as being at the apex of the food pyramid. In the case of birds of prey such as young Bonelli’s eagles (*Aquila fasciata*), there was evidence of *Campylobacter* detected in the nest [126] in around 11% of samples—together with *Salmonella* at around three times this level. Potentially worryingly, this included strains which showed antibiotic resistance.

### 5.3. Mammals

Domestic mammals have been described as a reservoir of *Campylobacter* spp. [17,20,177]. For example, *C. jejuni* and *C. coli* have been isolated from fecal samples of dogs and cats. Depending on the study, some examples show a higher prevalence as being described in dogs [178], but others show a higher value in cats [179]. *C. upsaliensis* has been found with a higher prevalence in dogs, principally in puppyhood and adolescent periods [180]. While the purpose of this paper is not to investigate domesticated species, they are mentioned here since feral dogs and cats have been shown to be infected in Australia, with principally *C. upsaliensis* and *C. jejuni* having been found in 11% and 4% of cats, respectively, whereas 34% of dogs carried *C upsaliesis*, 7% carried *C jejuni*, and 2% carried *C. coli* [181]. Moreover, it should be noted that even animals which are still pets are often not restricted to houses. In particular, cats are often allowed to roam freely in many countries and, although technically domesticated, have a number of similarities with those which are feral.

Rodents are another potential host group that can spread *Campylobacter* spp. Olkkola et al. [84] demonstrated that the highest prevalence occurred in yellow-necked mice (*Apodemus flavicollis*) and bank voles (*Myodes glareolus*) which carried *Campylobacter* spp. in 66.3 and 63.9% of the samples collected from these wild animals on farms and 41.5 and 24.4% of animals trapped from natural habitats, respectively. Kim et al. [81], in Korea over a 2-year period, captured house mice (*Mus musculus)* and harvest mice *(Micromys minutus)* which did not have any clinical symptoms. *C. jejuni* was only isolated from *M. minutus* (42/66, 63.6%). A single clone (MLST ST-8388) was found in all 42 *C. jejuni* isolates, and all isolates had the same virulence/survival-factor profile, except for the plasmid-mediated *virB11* gene. However, Sippy et al. [176] sampled voles (*Microtus* spp.), deer mice (*Peromyscus* spp.), house mice (*M. musculus*), brown rats (*Rattus norvegicus*), short-tailed shrews (*Blarina brevicauda*), least shrews (*Cryptotis parva*), eastern moles (*Scalopus aquaticus*), and other small mammals, but did not find any *Campylobacter* spp. in their samples. 

Bats have been detected as carriers of several zoonoses microorganisms. Adesiyun et al. [73] detected *Salmonella* spp. and *E. coli* in bats’ gastrointestinal tracts; however, *Campylobacter* was not present. Nevertheless, Hatta et al. [79] detected *C. jejuni* and *C. coli*, *C. helveticus*, *Campylobacter peloridis*, *Campylobacter insulaenigrae subantarcticus*, and *C. volucris* in Geoffroy’s Rousette (*Rousettus amplexicaudatus*) using high-throughput sequencing in rectal swab samples, suggesting that bats can be potential carriers of *C. jejuni*.

Other mammals have been considered as sources for infection. Mutschall et al. [83] identified raccoons (*Procyon lotor*) as ideal subjects for exploring the potential role that they play in the epidemiology of campylobacteriosis, because racoons can adapt to different environments, and live at the interface of rural, urban, and more natural environments. Briefly, in their study, they captured raccoons on five swine farms and five conservation areas in southwest Ontario, Canada. It was found that the prevalence of *Campylobacter* spp. in raccoon fecal samples was 46.3% (508/1096). Among the *Campylobacter*-positive raccoon samples, 502 (98.8%) were positive for *C. jejuni*, six (1.2%) for *Campylobacter* spp. (unidentified *Campylobacter* species), and one for *C. coli*. This suggested that raccoons may act as vectors in the transmission of clinically relevant *C. jejuni* subtypes at the interface of rural, urban, and more natural environments. Moreover, De Witte et al. [182] collected fecal samples of terrestrial zoo mammals from 6 different zoos in Belgium and observed both *Helicobacter* spp and unknown *Campylobacter*.

On the other hand, a cross-sectional study of the molecular epidemiology of *C. jejuni* in a dairy farmland environment [46] showed that 73.7% of wild rabbits (*Oryctolagus cuniculus*) can keep a similar genotype in cattle (the ST-21 complex), which is relevant to human infection. Rhynd et al. [85] demonstrated that asian mongooses (*Herpestes javanicus*) are carriers and shedders of *Salmonella* and *Campylobacter* spp. Moreover, Medley et al. [66] sampled fecal samples in humans, free-ranging banded mongooses (*Mungos mungo*) surface water, and river sediment samples in northern Botswana and reported *Campylobacter* spp. and *C. jejuni* as the main bacterium free-ranging banded mongooses (*M. mungo*). Also, *Campylobacter* spp. was widespread in humans with infections dominantly associated with *C*. *jejuni*; however, *Campylobacter* spp. was rare or absent in environmental samples, but half of the mongooses sampled tested positive (56%). The authors suggested that pathogen circulation and transmission in urbanizing wildlife reservoirs may increase human vulnerability to infection. 

In marine mammals, the prevalence of *Campylobacter* spp. has been described in captive and wild marine animals. De Witte et al. [182] isolated *C. insulaenigrae* in 1/11 seals and 3/6 sea lions in Belgian zoos. Greig et al. [78] detected *Campylobacter* spp. in 22/241 (9.1%) of harbor seals (*Phoca vitulina*), which included both wild and those caught after being stranded in Central California, USA. Meanwhile, Fooster et al. [183] detected *C. jejuni*, *C. coli*, *C. lari*, and *Campylobacter insulaenigrae* from 3 free-ranging harbor seals (*P. vitulina*) in Scotland, and Stoddard et al. [184] isolated *C. jejuni* (17/165, 10.3%), *C. lari* (5/165, 3%), and an unknown *Campylobacter* sp. (1/165, 0.6%) in elephant seals (*Mirounga angustirostris*) from Central California, USA. Moreover, Stoddard et al. [185] characterized 72 presumptive *C. lari* and unknown *Campylobacter* species strains using standard phenotypic methods, 16S rRNA PCR, and multilocus sequence typing (MLST). Baily et al. [75] isolated *C. jejuni* in wild-caught live grey seals (*Halichoerus grypus*), 24/50 dead and 46/90 live in the breeding colony on the Isle of May (Scotland). However, returning yearling animals (19/19) were negative for *C. jejuni*, suggesting the clearance of infection while away from the localized colony infection source. In addition, genome sequence was carried out, using a whole-genome multilocus sequence typing (MLST) as an approach to make a model of the genotype–host association. They demonstrated the spread of a human pathogen to a sentinel marine mammal species inhabiting a national nature reserve, probably through fecal contamination from agricultural land or human sewage [186].

## 6. Summary/Conclusions

It is clear that *Campylobacter* spp. can exist in domestic animals and the routes of the disease transmission have been described, although, for the purposes of this paper, they are only considered in the context of domesticated species which are existing as a feral lifestyle. Cumulatively, the various studies have led to an improvement in the understanding of the epidemiology using molecular approaches. Nevertheless, the dynamics of transmission between wildlife, domestic animals, and humans are still not fully clear yet. Moreover, the analytical approach (e.g., molecular versus cultural approaches) can lead to different infection levels being reported. For this reason, we have tended to place the emphasis on reporting species which can be infected, whilst trying to maintain an indication of the levels of infection. The level of disease present in wild populations is difficult to assess due to problems associated with finding diseased animals in the wild, as opposed to those either in captivity or those which have been domesticated.

This review has shown that wildlife can act as an important *Campylobacter* spp. reservoir, with several studies described in birds and mammals, but less in amphibians, reptiles, and fish. Although most *Campylobacter* studies have been carried out with either humans or domesticated animals, there are a number of studies which describe the potential roles of wildlife and the environment as a source of *C. jejuni* infection. In fact, not all studies have been related to human outbreaks with wildlife sources using whole-genome multilocus sequence typing (MLST). 

Fragmentation of landscape may influence human and animal exposure and *Campylobacter* infection dynamics, because anthropogenic resources can alter host–pathogen interactions, leading to either increased or decreased infection risk for wildlife and humans depending on the nature of provisioning and the particular host–pathogen interaction [23]. hen, it is necessary to understand the human-domestic animal–wildlife-environment interface. We have also included data on the relative level of incidence in different species, and this serves to demonstrate that different values were observed in different geographical areas. How much these differences vary may be down to the methods which were used to make assessments, geographical differences, or even temporal variation. Thus, the major purpose of this work was to identify the range of species in which members of the genus *Campylobacter* has been described.

In conclusion, wildlife animals such as birds, mammals, and reptiles can act as reservoirs of *Campylobacter* spp., and they play an important role in the transmission of these bacteria. However, a few studies have shown evidence that *Campylobacter* can either be transmitted to humans or animals can be an important host to transmission. It is important to carry out more studies of the role played by wildlife, mainly birds, as well as other wild animals and the interface with domestic animals and humans. This is particularly true given the number of countries where no research has been carried out on the presence of *Campylobacter* spp. in wild, or even feral, vertebrate species. However, we anticipate that as the number of species investigated increases, the true extent of infection will become even clearer.

## Data Availability

Not applicable.

## Supplementary Materials

The following supporting information can be downloaded at: https://www.mdpi.com/article/10.3390/ani13081334/s1, Table S1: Epidemiological wildlife studies carried out with *Campylobacter* spp.
